# Cardiovascular magnetic resonance imaging in rheumatic heart disease

**Published:** 2018

**Authors:** Ntusi NAB

**Affiliations:** Division of Cardiology, Department of Medicine, University of Cape Town and Groote Schuur Hospital; Cape Universities Body Imaging Centre, Faculty of Health Sciences, University of Cape Town; Hatter Institute of Cardiovascular Research in Africa, Department of Medicine, University of Cape Town, South Africa

Rheumatic heart disease (RHD), a sequela of pharyngeal and skin infection with group A β–haemolytic Streptococcus, affects approximately 33 million persons globally, with low– and middleincome countries (LMICs) disproportionately more affected.^1^ RHD globally contributes the largest share to cardiovascular mortality in individuals under 50 years old.^2^ Sub–Saharan Africa (SSA) bears the greatest burden of cardiovascular morbidity and mortality related to RHD.^3^ In highly endemic parts of SSA, the prevalence of RHD ranges from 4.6 to 21.7 per 1 000 individuals, based on echocardiographic screening.^4^

Complications secondary to RHD cause disability–adjusted life–years (DALYs) of 142.6 per 100 000 individuals globally, translating to 0.43% of total global DALYs.^1^ The rate of DALYs attributable to RHD is highest in SSA, where it negatively affects young and economically active members of the population.^5^

**Fig. 1:**
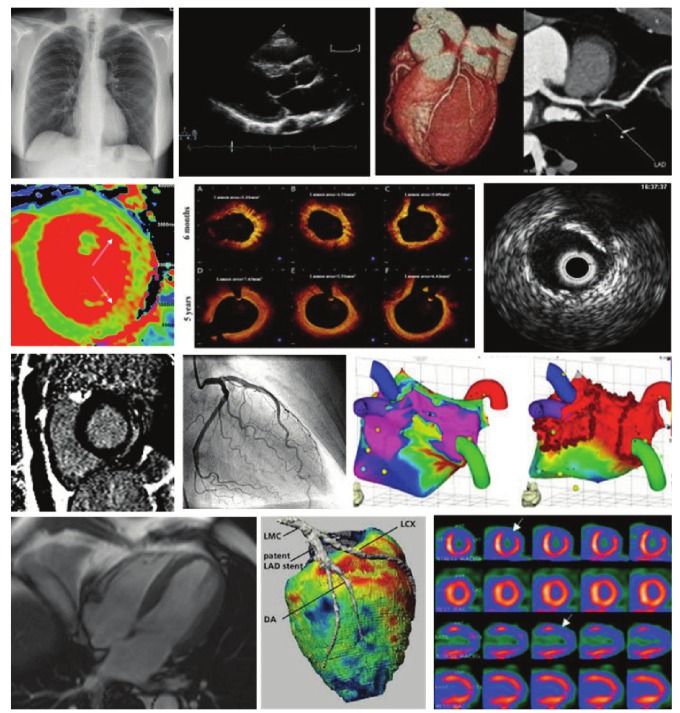
Different modalities of cardiovascular imaging.

**Fig. 2:**
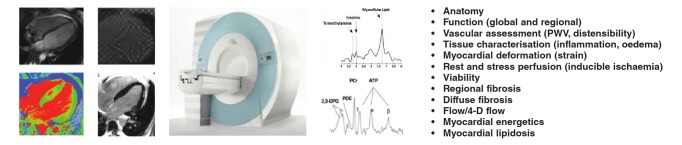
Multiparametric capability of CMR.

Multiple cardiovascular imaging modalities are important for the assessment of CVD and many are entrenched into the modern practice of cardiovascular medicine (Fig. 1). Cardiovascular magnetic resonance (CMR) is the gold–standard technique for many indications. It permits, in a single examination, comprehensive characterisation of functional, morphological, metabolic, tissue and haemodynamic sequelae of cardiovascular pathologies (Fig. 2).^6^ The high spatial and temporal resolution of CMR, coupled with excellent tissue contrast enables complete assessment of multiple parameters without exposure to ionising radiation. Further, the ability to obtain images in any tomographic plane regardless of body habitus confers significant advantage in patients with limited sonographic acoustic windows.

CMR creates images from atomic nuclei with uneven spin using radiofrequency pulses in the presence of a powerful magnetic field. Hydrogen, which is abundant in fat and water, is the most commonly used atom for MR imaging; and tissue contrast in CMR is accounted for by three important parameters: T1 and T2 relaxation and proton density. CMR is safe, especially when compared with X–ray–based techniques. The main MR contrast agent, gadolinium, has been shown to be safe in millions of patients who have received it over decades, and nephrogenic systemic sclerosis has not been reported with the newer macrocyclic gadolinium–based agents, which are preferentially used in patients with renal dysfunction due to the high risk posed by iodinated contrast agents.^6^,^7^

Characterisation of myocardial tissue is a unique feature of CMR, traditionally achieved through late gadolinium enhancement (LGE) imaging, and based on the relative difference in volume of distribution of intravenously administered gadolinium [and subsequent alteration of longitudinal relaxation (T1) times] between normal and abnormal myocardium.^8^ Hence, LGE–CMR permits identification of focal fibrosis. More recently, native (pre–contrast) T1 and T2 mapping techniques have allowed direct measurement of myocardial relaxation times on a pixel–wise basis, parameters which have been extensively validated, offering similar diagnostic performance and superior sensitivity for inflammation, infiltration, acute injury and fibrosis, compared with delayed enhancement imaging in detecting myocardial pathology.^9^ Post–contrast T1 mapping and estimation of the extracellular volume (ECV) allow for the assessment of the degree of diffuse myocardial fibrosis.^10^

Echocardiographic studies of RHD are established in clinical practice and are indispensable for the comprehensive assessment of valve lesions secondary to RHD, through confirmation of aetiology of the valvular lesion and exclusion of non–rheumatic causes of valve lesions. M–mode and two–dimensional crosssectional echocardiography are important for the assessment of chamber size and function, diastolic dysfunction, valve morphology and function, and both atrial and myocardial remodelling. Colour–flow Doppler evaluates flow across valves and can assess the haemodynamic effects of both stenotic and regurgitant lesions.

Furthermore, serial echocardiography is important for monitoring of disease progress as well as efficacy of surgical repair or replacement. The use of strain imaging and threedimensional echocardiography is important for risk stratification and in planning and predicting surgical outcomes.^11^ Portable echocardiography plays a crucial role in screening for RHD and is important for defining disease burden, clarifying referral pathways and informing policy for scaling up RHD control programmes. The publication of the World Health Federation criteria for the echocardiographic diagnosis of RHD in 2012 has provided standardisation and improved both specificity and utility of echocardiographic screening for RHD.^12^

**Fig. 3:**
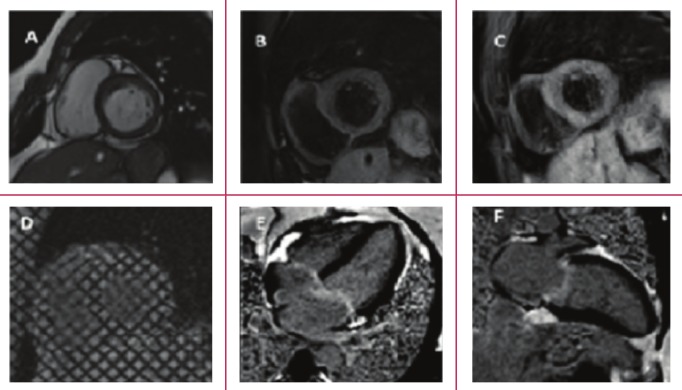
Patient with heart block and acute myocarditis confirmed to be due to acute rheumatic fever. A. balanced steady–state freeprecession image showing a short–axis ciné; B. T1–weighted image showing increased myocardial signal–intensity ratio; C. T2–weighted imaging showing increased myocardial signal–intensity ratio (in keeping with myocardial oedema); D. Ciné tagging imaging of the short axis confirming impaired circumferential strain and strain rate; E. Horizontal long–axis (fourchamber) LGE image showing no myocardial enhancement but evidence of valvulitis, with valvular and atrial enhancement; F. Vertical long–axis (two–chamber) LGE image confirming lack of myocardial LGE, but mitral valve enhancement.

CMR has been demonstrated to be capable of performing many of the applications described above.^13^ Multiparametric CMR has been employed in small case series and case reports in the diagnosis and guidance of management of patients with RHD. In a series of three patients with chronic RHD, CMR was found to be associated with LGE in atrial walls.^14^ Using dated CMR sequences as a comparator, Mutnuru et al. found echocardiography to be a more reliable tool for diagnosis of RHD.^15^ Our group has recently reported on the role of CMR in unravelling the pathophysiology of heart block and myocarditis in a patient subsequently confirmed to have acute rheumatic fever (Fig. 3).^16^

Edwards and colleagues reported on a CMR cross–sectional study of 35 patients (mean age 60 years) with asymptomatic moderate and severe primary degenerative mitral regurgitation but impaired VO2 max and found dilated left ventricular (LV) volumes, preserved LV systolic function, evidence of impaired longitudinal and circumferential strain, LGE in 30% of subjects, and evidence of diffuse myocardial fibrosis from elevated ECV.^17^ The authors concluded that patients with moderate to severe mitral regurgitation have increased myocardial fibrosis, impaired myocardial strain and reduced exercise capacity.

In this issue of the journal (page 150), Meel and colleagues report, similarly, on a study of 22 patients with chronic rheumatic mitral regurgitation and 14 age– and gender–matched controls characterised by echocardiography, LGE–CMR (for assessment of focal fibrosis) and serum biomarkers of collagen turnover.^18^ The key findings were that 18% of the patients had evidence of LGE, while none was observed in the controls. As expected, on both CMR and echocardiography, patients with rheumatic mitral regurgitation had greater LV dimensions and greater LV mass, though overall LV systolic function was not different. Procollagen IC peptide (PIP) and procollagen III N–terminal pro–peptide (PIIINP) were similar between patients and controls, however, matrix metalloproteinase–1 (MMP–1) activity was increased in the patient group. The authors concluded that chronic rheumatic mitral regurgitation is characterised by the predominance of collagen degradation rather than increased synthesis and myocardial fibrosis.

RHD is characterised by chronic inflammation which, in many other inflammatory cardiac conditions, 6,8–10 is associated with frequent focal myocardial fibrosis. In this small study, the authors reported the incidence of myocardial fibrosis on LGE–CMR at 18%. It would have been instructive if the investigators had utilised native T1 mapping or ECV to assess for the presence of diffuse myocardial fibrosis in RHD, which in my experience is found commonly. The authors attribute the infrequent LGE to increased expression of biomarkers of collagen degradation. However, the relationship of serum biomarkers of collagen synthesis, which have low specificity for cardiac fibrosis, has been inconsistently reported in the literature, with a prior publication reporting increased biomarkers of collagen synthesis in RHD.^19^

In the future, CMR may play an increasingly important and complementary role to echocardiography in the evaluation and management of patients with RHD. An advantage of CMR over echocardiography in RHD is the ability to provide accurate and reproducible information on tissue characteristics, including myocardial fibrosis and oedema, without dependence on the presence of adequate acoustic windows and operator experience.^5^ CMR may provide additional diagnostic information when echocardiographic imaging is suboptimal. However, the utility of CMR for the evaluation of RHD may be limited by its relative inability to be used in patients with certain types of metallic implants, local artefacts from prosthetic valves, lack of availability, expense of the tests and limited expertise in RHD–endemic countries. Furthermore, data regarding prognostic significance of CMR in RHD are lacking.^5^
